# Focal Epilepsy Associated with Glioneuronal Tumors

**DOI:** 10.5402/2011/867503

**Published:** 2011-07-07

**Authors:** Giulia Loiacono, Chiara Cirillo, Francesco Chiarelli, Alberto Verrotti

**Affiliations:** Department of Paediatrics, University of Chieti, 66100 Chieti, Italy

## Abstract

Glioneuronal tumors are an increasingly recognized cause of partial seizures that occur primarily in children and young adults. Focal epilepsy associated with glioneuronal tumors is often resistant to pharmacological treatment. The cellular mechanisms underlying the epileptogenicity of glioneuronal tumors remain largely unknown. The involved mechanisms are certain to be multifactorial and depend on specific tumor histology, integrity of the blood-brain barrier, characteristics of the peritumoral environment, circuit abnormalities, or cellular and molecular defects. Glioneuronal tumors presenting with epilepsy were observed to have relatively benign biological behavior. The completeness of the tumor resection is of paramount importance in avoiding tumor progression and malignant transformation, which are rare in cases of epileptogenic glioneuronal tumors. An evolving understanding of the various mechanisms of tumor-related epileptogenicity may also lead to a more defined surgical objective and effective therapeutic strategies, including antiepileptogenic treatments, to prevent epilepsy in at-risk patients.

## 1. Introduction

Glioneuronal tumors are an increasingly recognized cause of partial seizures that occur primarily in children and young adults [1, 2].

These are tumors with an admixture of glial and neuronal components. Both cell types are thought to be part of the same neoplastic process. Entrapment of preexisting neurons by an infiltrating glioma therefore has to be distinguished from glioneuronal tumors. More well-established examples of glioneuronal tumors include dysembryoplastic neuroepithelial tumors (DNTs) ganglioglioma and desmoplastic infantile ganglioglioma. More recently recognized entities partly included in the latest version of the WHO classification include the rosette-forming tumor of the fourth ventricle the papillary glioneuronal tumor and rosetted glioneuronal tumor/glioneuronal tumor with neuropil-like islands. The glial component in these tumors varies but often resembles either a pilocytic astrocytoma or an infiltrating glioma with astrocytic or oligodendroglial features [3].

Gangliogliomas and DNTs arise most commonly in the temporal lobe and appear to be associated with an increased incidence of cortical dysplasia or neuronal migration abnormalities [1, 4, 5]. 

Focal epilepsy that is often resistant to pharmacological treatment is a common presenting symptom of glioneuronal tumors [1, 2]. Even though the biological behavior of these tumors is usually benign, especially when patients present only with epilepsy, cases of tumor progression or malignant transformation have been reported [1, 6, 7].

While a subset of epilepsy patients with glioneuronal tumors may be candidates for epilepsy surgery, a better understanding of underlying mechanisms of epileptogenesis in this group of developmental brain disorders could lead to more effective therapeutic strategies, including antiepileptogenic treatments to prevent epilepsy in at-risk patients.

## 2. Epileptogenesis

The cellular mechanism(s) underlying the epileptogenicity of glioneuronal tumors remain largely unknown. The high success rate of “lesionectomy” during epilepsy surgery, with many studies reporting over a 60–75% seizure-free rate, also supports the idea that the lesions directly produce seizures [8–10]. However, this still leaves a substantial minority of patients that continue to have seizures following lesionectomy, suggesting that the epileptogenic zone was not contained within the lesion in those cases. Furthermore, the success of lesionectomy in eliminating seizures may have other interpretations: the margins of resection typically contain some “normal” perilesional tissue, which may actually be the primary source of the seizures. Alternatively, perilesional cortex, immediately adjacent to or even distant from the lesion, may generate the seizures, but may be somehow dependent on the lesion for epileptogenesis. Another controversial issue is whether epileptogenesis in malformations of cortical development is primarily due to circuit abnormalities or cellular and molecular defects [11]. 

A growing intracranial lesion can both structurally and functionally alter the surrounding brain tissue with edema, vascular insufficiency, inflammation, and release of metabolically active molecules, hence also promoting seizure activity. The involved mechanisms are certain to be multifactorial and depend on specific tumor histology, integrity of the blood-brain barrier, and characteristics of the peritumoral environment [12].

Seizures clearly consist of synchronous electrical activity reverberating through complex neuronal networks and thus ultimately must always include abnormalities on the circuit level. Ultimately, both network and cellular/molecular abnormalities will stimulate epileptogenesis by upsetting the normal physiological balance between excitation and inhibition in the brain [11].

An important role in hyperexcitability has been attributed to the neuronal component of these lesions, consisting of highly differentiated cells containing, for example, neuropeptides, neurotrophins, gap junctions, and receptors for different neurotransmitters [2, 8, 13–15]. Neuron-glia interactions may also play a critical role in the generation of seizures. Accordingly, several studies demonstrate alterations of functional properties of glial cells, involving plasma membrane channels and receptors that might be involved in epileptogenesis [16]. A recent hypothesis is that the presence of activated astrocytes and microglia in focal cortical dysplasia and glioneuronal tumors may be related to the epileptogenicity of these lesions through the production of inflammatory cytokines [17, 18]. Particular attention has been focused on the role of the interleukin-1*β*- (IL-1*β*-) activated pathways in ictogenesis. Intracerebral application of IL-1*β* in rodents prolongs seizure activity [19]. Accordingly, intracerebral application of the naturally occurring antagonist of the IL-1 receptor (IL-1Ra) mediates powerful anticonvulsant effects [18]. Inhibition of the production of the biologically active form of IL-1*β* using blockers of interleukin-converting enzyme, also named caspase-1, significantly reduces seizures in rodents [20]. These observations strongly support a proconvulsant role of IL-1*β* produced in the brain in pathological conditions, such as during seizures or as a consequence of underlying inflammatory processes [19, 21]. It has been shown also that the density of activated microglia (one major source of cytokines in the brain) in focal cortical dysplasia and glioneuronal tumors correlates with both the duration of epilepsy and the frequency of seizures [22, 23]. Finally, genetic studies reported a polymorphism in the promoter region of the IL-1*β* gene, which is associated with therapy-resistant temporal lobe epilepsy and in children with febrile seizures [24]. Ravizza et al. [16] demonstrated that chronic inflammatory reactions are intrinsic to lesional tissue and sustained by aberrant cells and the recurrence of seizures in lesional tissue likely contributes to perpetuate inflammation. 

Particular attention has been focused on the neuronal glutamatergic population, consisting of highly differentiated cells expressing different glutamate receptor subtypes [25]. However, compelling evidence in human epileptogenic tissue and in different experimental models of seizures indicates that anatomical and functional alterations of the GABAergic networks may also critically contribute to epileptogenesis. Dysfunction of the inhibitory system may result from loss of the GABAergic interneurons [26]. Some findings suggest that alterations in GABA-mediated synaptic inhibition in malformations of cortical development could be secondary to reductions in GABA transporter expression [27, 28] or changes in the expression and/or function of specific GABA receptor subtypes [29, 30]. Aronica et al. [31] suggest that the cellular distribution of components of the GABAergic system in low-grade gangliogliomas, together with the perilesional changes, suggests that alterations of the GABAergic system may contribute to the complex abnormal functional network of these highly epileptogenic developmental lesions.

The stem cell epitope CD34 is highly expressed in gangliogliomas. The presence of a prominent CD34-immunoreactive cell element in glioneuronal lesions associated with focal epilepsies points towards an origin from dysplastic and/or neoplastically transformed precursor cells [32]. Several studies [2, 32–34] have noted the ability of CD34, in highlighting a distinct subset of small cells with ramifying processes, in seizure-associated focal cortical dysplasia, neoplastic lesions like gangliogliomas and pleomorphic xanthoastrocytomas, thus aiding in differentiating them from other low-grade neoplasms. CD34 is a glycophosphoprotein, normally expressed on the surface of haematopoietic progenitors of myeloid series, on vascular endothelium, and on developing central nervous system [35]. Although the exact nature of these CD34-immunopositive cells is not known, it has been conjectured that they represent a form of glioneuronal progenitor cell, as some have been found to colocalize with traditional neuronal markers [34]. Studies have suggested that CD34 has a probable role in cell adhesion and intracellular signal transduction pathways [36]. Recent reports [37] have shown that ex vivo bone marrow CD34+ cells express neuronal and oligodendroglial markers or develop into microglial cells after migrating into the brain. The fact that these CD34+ cells also express transcription factors (that are found during early development) elicits the hypothesis that they may be pluripotent embryonic-like stem cells [37]. A recent study [38] established a role of CD34 as a diagnostic marker in glioneuronal lesions associated with epilepsy, especially in dual lesions of gangliogliomas with focal cortical dysplasia, but also indicates a possible pathogenetic relationship between focal cortical dysplasia and gangliogliomas, while suggesting a different aetiology for DNT.

Some studies point to a role of genes and pathways in epilepsy-associated glioneuronal lesions [39, 40], otherwise associated with rare familial disorders such as tuberous sclerosis complex (TSC) [41]. TSC is frequently caused by germline mutations of the TSC1 (hamartin) and TSC2 (tuberin) genes on chromosomes 9q and 16p, respectively. However, mutations in TSC1 or TSC2 are only found in 85% of the TSC patients [42]. Hamartin and tuberin constitute a tumor suppressor complex operating as key factor in the PI3K/mTOR pathway, which is involved in cell size control, cell adhesion/migration, and cell fate determination [39]. Although recent data suggest PI3K pathway activation in focal cortical dysplasia, gangliogliomas, and TSC-associated lesions, distinct patterns of activated elements within the PI3K-signal transduction cascade are observed between focal cortical dysplasia and cortical tubers in TSC. Tuberin has been also shown to constitute a chaperon for hamartin, and its impaired availability may contribute to aberrant cellular distribution of the complex [43]. Aberrant expression of PI3K-pathway components (ezrin, radixin, and moesin) in focal cortical dysplasia and gangliogliomas was observed [44]. These alterations may relate to compromised interactions of dysplastic cellular components in epilepsy-associated glioneuronal lesions and be involved in aberrant PI3K-pathway signaling in epilepsy-associated malformations.

Tyrosine kinase receptors trkA, -B, and -C were observed to be strongly expressed in neuronal components of gangliogliomas, focal cortical dysplasia and DNET [15]. Fassunke et al. [45] showed NGFR as the most abundant transcript in gangliogliomas and suggested gangliogliomas and DNETs to share some differential expression patterns, that is, reduced expression of NELL2, PRKCB1, HSJ2, and ARF3; however, they identified several gene expression patterns that were specifically altered only in gangliogliomas such as for TRIB1, ST6GalNac4, and LDB2, related to distinct dysplastic features of these glioneuronal tumours.

The plasminogen (fibrinolytic) system comprises the inactive proenzyme, plasminogen, that can be converted to the active enzyme plasmin by two serine proteases, the tissue-type plasminogen activator (tPA) and the urokinase-type plasminog activator (uPA) [46]. In the CNS, increasing evidence suggests a crucial role of the plasminogen system in a broad range of physiological and pathological processes ranging from neuronal development, cell migration, and invasion, cell growth and apoptosis, immune responses, inflammation, angiogenesis, and regulation of synaptic remodeling and neuronal plasticity [47, 48]. Several experimental findings identified a role for tPA in the mechanisms underlying seizure activity [48, 49]. Interestingly, induction of plasminogen activators (PAs) has been observed in different experimental models of epilepsy [50], and gene expression profile analysis of gangliogliomas revealed that both tPA and uPA represent one of the most upregulated genes in these epileptogenic lesions. Recently, the cellular distribution of uPA and uPAR has been characterized in rat hippocampus during epileptogenesis [50]. Iyer et al. [51] demonstrated that activation of the plasminogen system, as exemplified by increased expression of tPA and uPA, indeed occurs in human focal chronic refractory epilepsy, confirming the studies performed in experimental models of temporal lobe epilepsy. The chronic expression of PAs together with the upregulation of uPAR might contribute to the mechanisms underlying the epileptogenicity of focal lesions, through direct modulation of neuronal activity or, indirectly, through regulation of the inflammatory response and/or epileptogenic tissue remodeling. Several mechanisms are possibly involved, including a direct regulation of neuronal excitability via NMDA receptors [48]. Additionally, both tPA and uPA may contribute to the disruption of blood-brain barrier integrity and amplification of inflammatory infiltrates [52], which have been recently shown to play a critical role in chronic refractory epilepsy [17, 53]. Finally we have to consider the key role of the PA system in the complex tissue remodeling that occurs during epileptogenesis, such as axonal reorganization, angiogenesis, and astrogliosis [54].

The resistance to the pharmacological treatment with a broad range of antiepileptic drugs (AEDs) is characteristic of these developmental lesions [1, 55]. The mechanisms underlying this multidrug resistance are still elusive. One possible mechanism to account for this medical intractability is the inadequate drug concentration in the epileptogenic areas. In the last years attention has been focused on multidrug transporters, such as P-glycoprotein (P-gp) and the family of multidrug resistance-associated proteins. Several reports indicate that common AEDs, such as carbamazepine, phenytoin, and valproate, are substrate of these multidrug transporters [56, 57]. Thus, changes in the expression of both P-gp and multidrug resistance-associated proteins (MRPs) may critically regulate the extracellular brain concentration of a broad range of AEDs, contributing to the pharmaco-resistance. The overexpression of multidrug transporter protein is a common feature of both focal cortical dysplasia and gangliogliomas [55]. This overexpression is likely responsible for, or at least contributes to, the intrinsic drug resistance of these developmental lesions. The cell-specific expression of MRP1 and P-gp would impair drug responsiveness at three consecutive steps: (1) at the level of the endothelial cells in lesional capillaries (P-gp), leading to a decreased tissue drug concentrations; (2) at the level of the astrocytic end-foot processes surrounding brain capillaries and neurons (P-gp and MRP1), promoting a flux from brain extracellular space back to the bloodstream, thus reducing the drug available for interaction with its targets in neurons; (3) at the level of the neuronal and neuroglial epileptogenic component of the lesion, interfering with the intracellular activities of the AEDs. Several inhibitors of P-gp and, more recently, of MRPs are available and are currently in the clinical trial stage in human cancer patients [58]. Thus, evaluation of the possible use of these inhibitors as adjunctive treatment in pharmaco-resistant epilepsy is certainly worthwhile.

## 3. Treatment

Epilepsy that is often resistant to pharmacological treatment is a common presenting symptom of glioneuronal tumors (in 75% of cases), and the temporal lobe is the most frequent location (in 60% of cases). The best approach to management of epilepsies related to benign tumours is controversial. Several studies have suggested a very benign clinical course in most patients with gangliogliomas. However, tumor recurrence, malignant progression, and secondary glioblastoma multiforme are observed in some patients [2, 5, 59]. Malignant transformation has been associated with incomplete tumor resections and radiation therapy [5, 60].

Epilepsy control represents the main therapeutic goal [1, 2, 5]. Findings from several studies have indicated that early surgery for children with the forms of epilepsy associated with primary brain tumors can improve the patient's psychosocial and intellectual development, reduce the incidence of secondary epileptogenesis, ensure an accurate diagnosis, and prevent the malignant transformation of otherwise benign tumors [1, 2, 61, 62]. 

Controversy exists regarding the most appropriate surgical treatment for children suffering from epilepsy associated with brain tumors. Some investigators consider complete tumor excision sufficient [62–64] whereas others advocate the identification and resection of epileptogenic zones as necessary to provide a good seizure outcome [1, 5, 59, 65].

Few studies, however, have specifically focused on the long-term seizure outcome after lesionectomy in selected series of children harboring glioneuronal tumors. Other authors have investigated seizure outcome after lesionectomy in groups in which glioneuronal tumors occurred together with other different epileptogenic types of tumor [62, 63].

Giulioni et al. [66] demonstrated that lesionectomy results in good seizure control in the majority of children with epileptogenic glioneuronal tumors. 

Recently Yang et al. [67] reviewed the modern literature regarding the effects of early surgical intervention on the clinical outcome of patients with ganglioglioma: seizure control was significantly improved when surgical intervention occurred less than 3 years after symptom onset (78% versus 48%), thus an early surgical intervention is significantly associated with improved clinical seizure control.

Furthermore, glioneuronal tumors presenting with epilepsy were observed to have relatively benign biological behavior. The seizure outcomes do not appear to be related to the duration of epilepsy, seizure type, and frequency or completeness of tumor removal. The completeness of the tumor resection is of paramount importance in avoiding tumor progression and malignant transformation, which are rare in cases of epileptogenic glioneuronal tumors [66].

Finally, despite the probable developmental defects in glioneuronal proliferation in the relevant malformations of cortical development, classes of drugs are already available that can inhibit cell proliferation. In particular, the recent discovery of mTOR pathway dysregulation in TSC, as well as focal cortical dysplasia and gangliogliomas, offers a remarkably direct “bench-to-bedside” strategy for treating these malformations of cortical development. Rapamycin will specifically inhibit the mTOR pathway, leading to decreased cellular proliferation [42], and has already been tested in clinical trials for tumor growth in TSC [68].

## 4. Conclusion

Patients with drug-resistant epilepsy associated to glioneuronal tumors should undergo evaluation in dedicated epilepsy surgery programmes. The procedure should include diagnosis of the type of tumour by advanced imaging and radio-nuclear methods followed by rapid determination of intractability to antiepileptic drug treatment. Although many patients become seizure-free with a standard “lesionectomy,” a significant proportion of patients continue to have seizures despite lesionectomy and might benefit from a more extensive surgical resection involving broader perilesional regions. A large-scale screening of currently available drugs have identified a number of drugs that can upregulate astrocyte glutamate transporters, which may counteract the detrimental effects of impaired astrocyte glutamate transport seen in epilepsy and other neurological disorders [11]. Glial-selective drugs may also have the benefit of fewer neurological and cognitive side effects than one would expect with a comparable drug affecting neurons. 

Future advances in imaging techniques and molecular markers might be able to more accurately identify the true extent of the epileptogenic zone that needs to be resected to achieve seizure freedom. Further advances into the distinct pathophysiologies of epilepsy associated with different tumor histologies will promote an even more rational choice of medical therapy. 

An evolving understanding of the various mechanisms of tumor-related epileptogenicity and the role of the perilesional tissue may also lead to a more defined surgical objective for the patient presenting with tumor-related epilepsy. In addition to pharmacological interventions, future therapeutic gene replacement or gene manipulation approaches may eventually allow a more definitive correction of the molecular genetic defects in these diseases.
